# Orthopaedic Surgeon Communication Skills: Perception of Empathy and Patient Satisfaction Through the Use of Anatomic Models

**DOI:** 10.5435/JAAOSGlobal-D-18-00071

**Published:** 2018-11-02

**Authors:** Edwin L. Portalatín, Luis F. Carrazana, Roberto Colon, Ricardo Abreu, Dennys Rivera, Luis Lojo

**Affiliations:** From the Department of Orthopaedic Surgery (Dr. Portalatín, Dr. Carrazana, Dr. Colon, and Dr. Lojo), University of Puerto Rico, Medical Science Campus, and the Orthopaedic Department, University of Puerto Rico, Medical Science Campus (Dr. Abreu and Rivera), San Juan, Puerto Rico.

## Abstract

**Introduction:**

Patient satisfaction is an increasingly emphasized measure of patient-centered care and important component of reimbursement programs. Orthopaedic surgeons are regarded as low-empathy surgeons. Our goals were to understand the role of anatomic models during the orthopaedic appointment and how their use can affect patient satisfaction and perceived empathy.

**Methods:**

New patients at an outpatient clinic were asked to participate in a postencounter questionnaire to asses empathy perception (n = 304). Clinic days were randomly assigned to use anatomic models during the encounter to assist with clinical information transmission. The instrument provided contained Consultation and Relational Empathy questionnaire (ie, a person-centered process that was developed to measure empathy in the context of the therapeutic relationship during a one-on-one consultation between a clinician and a patient).

**Results:**

A total of 304 participants were included in the study. Analyses of the sociodemographic characteristics did not reveal any significant difference between the control and experimental groups. Consultation and Relational Empathy scores for the nonanatomic group (46.0 ± 9.0) and anatomic group (48.0 ± 7.7) were not statistically different (*P* = 0.482). The encounter time was significantly increased with the use of anatomic models (*P* < 0.005).

**Discussion:**

The use of anatomic models during initial orthopaedic encounter did not improve perceived empathy and satisfaction scores in our study. Longer encounter time in the orthopaedic appointment does not mean higher empathy perception.

**Conclusion:**

Orthopaedic surgeons have the duty to find new strategies to improve communication with the patient. Better communication has been associated with better patient satisfaction. Further investigation should be considered to use other strategies to provide better care for our patients.

Patient satisfaction is an increasingly emphasized measure of patient-centered care and important component of reimbursement programs.^1^ Empathy demonstrates the physicians understanding of and concern about the patient's thoughts and feelings.^4^ Patient-rated surgeon empathy was the strongest driver of patient satisfaction.^1^ The literature suggests that a decline in empathy exists that begins during clinical years of medical school, which continues throughout residency training.^6^ In a study by Tongue et al, 75% of the orthopaedic surgeons felt that they communicated satisfactorily with their patients, but only 21% of the orthopedic patients reported having adequate communication with their caretakers.^4^

Surgeons need to conduct conversation about complicated medical issues, treatment choices, complexities of surgical procedures and options, and they have to allay patient's fears and build trust during short visits.^3^ Consequently, surgeons require sophisticated skills in a variety of communication tasks, including exchanging information, responding to patients' emotions, and engaging in informed and collaborative decision making.^3^ Breakdowns in communication between physicians and patients lead to patient anger, dissatisfaction, and possible litigation.^5^ Surgeons are particularly susceptible to the decline in empathy as a byproduct of the nature of their work.^6^ If physicians listen for 2 minutes, the patient will tell you 80% of what needs to be known.^4^ Lower surgeon empathy was predictive of patient-perceived surgeon rush.^1^

Orthopaedic surgeons are regarded as high-tech low-empathy physicians. The purpose of this study is to analyze the use of anatomic models during the orthopaedic appointment. Patients will grade the appointment with Consultation and Relational Empathy (CARE) Measure, an instrument designed and validated to measure patient satisfaction and empathy. CARE bases the measure in process rather than outcomes and provides doctors with direct feedback of their strengths and weaknesses in terms of relational empathy, as perceived by their patients.^15^ Currently no literature exists which advocates in the use of anatomic models during the orthopaedic appointment and how their use can affect patient satisfaction and perceived empathy.

## Methods

After approval by our institutional review boards, the study was conducted at our institution outpatient clinics. Clinic days were randomly assigned to use anatomic models or not use anatomic models in all the new patients who had an appointment. The anatomic model was used during the encounter to communicate clinical information such as but not limited to pathophysiology, prognosis, treatment, and complications associated with their condition. Resident and attending physicians were included as primary communicators in the encounter and were responsible to record the encounter time.

After the encounter was culminated, patients were asked to participate in an anonymous survey pertaining their appointment. If patients agreed to participate, informed consent was obtained, and they were taken to a private room to complete the survey. The control group included patients who had a medical appointment without the use of anatomic models to explain their condition, prognosis, treatment options, and follow-up. The anatomic model group included patients in which their medical appointments occurred with the use of anatomic models to explain their condition, prognosis, treatment options, and follow-up.

### Patient Selection

Participants were older than 21 years and had the capacity to consent and read. Only new patients at our clinics, defined as first timers in our institution regardless if patients had evaluations at other institutions, were invited to participate in the study. Exclusion criteria included patients who already had surgical treatment for the condition evaluated. The study contains 304 participants who meet the inclusion criteria.

### Instrument

The first part of the instrument contains a sociodemographic survey. Participants were asked to provide their age, civil status, type of medical insurance, and level of education. Similarly, patients were also asked to describe whether their chief report was associated with trauma. Upon completion of the sociodemographic survey is completed, the instrument will contain questions to evaluate empathy and satisfaction perception.

**Figure 1:**
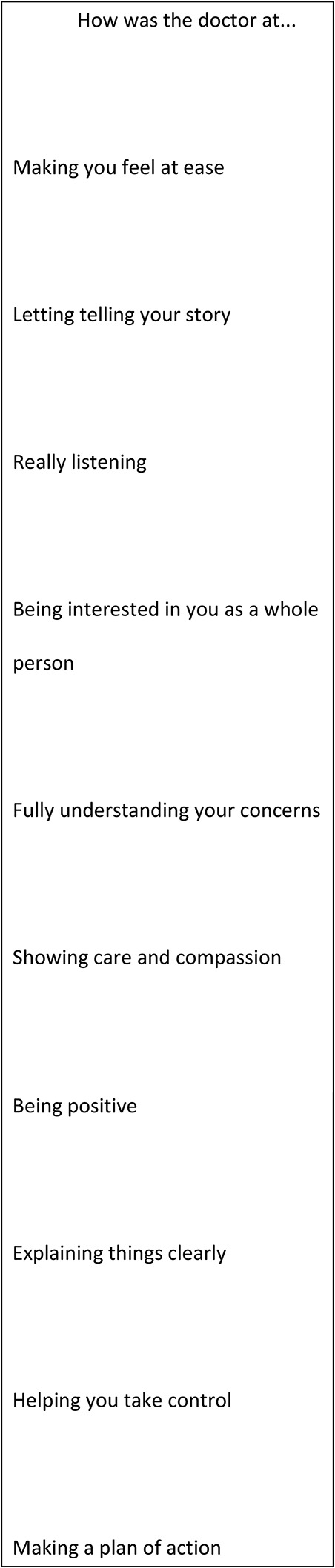
Table showing Consultation and Relational Empathy Measure questions. (Reproduced with permission from Mercer SW, Maxwell M, Heaney D, Watt GC: The consultation and relational empathy measure: Development and preliminary validation and reliability of an empathy—based consultation process measure. *Family Practice* 2004;21:699-705.)

**Figure 2:**
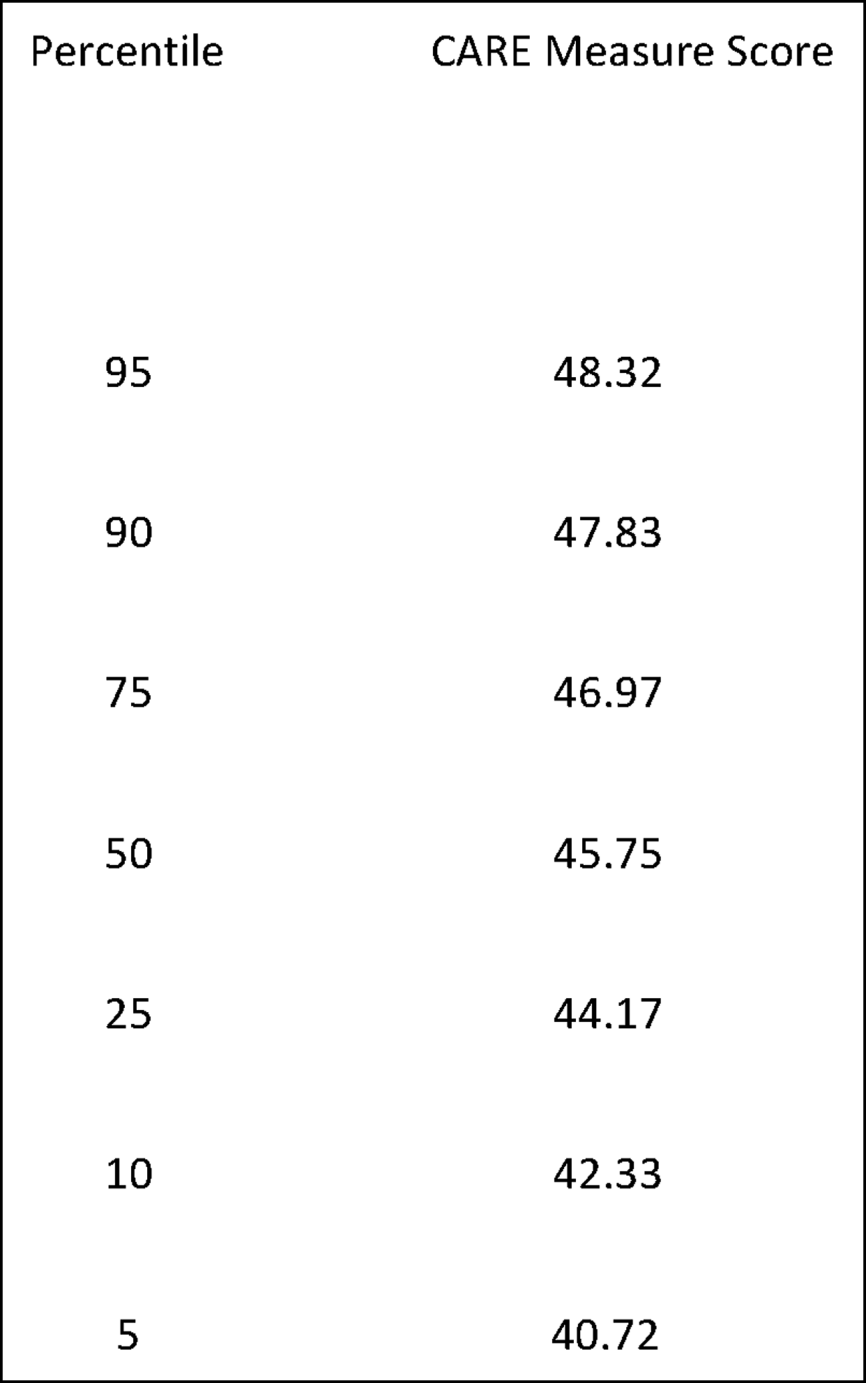
Table showing Consultation and Relational Empathy Measure normative values. (Reproduced with permission from Mercer SW, McConnachie A, Maxwell M, Heaney D, Watt GC: Relevance and practical use of the consultation and relational empathy (CARE) measure in general practice. *Family Practice* 2005;22:328-334.)

The CARE Measure is a questionnaire developed as a person-centered process to measure empathy in the context of the therapeutic relationship during a one-on-one consultation between a clinician and a patient. The CARE Measure consists of 10 items (Figure 1) related to patients' perception of physicians understanding of and response to their concerns.^2^ Results provide physicians a direct feedback of their relational empathy from the patients' perspective.^6^ The 10 questions will be evaluated on a scale of poor, regular, good, very good, or excellent. The validity and reliability of the CARE Measure has been demonstrated in various studies.^7,8^ CARE Measure normative values for interpretation of results are presented in Figure 2.

### Data and Statistical Analysis

The data collected were tabulated using the Microsoft Excel program. Overall summary statistics of patient baseline characteristics were calculated in terms of mean values and SDs for continuous variables and percentages and frequencies for discrete variables. The scoring system for each item in the CARE instrument is “poor” = 1, “fair” = 2, “good” = 3, “very good” = 4, and “excellent” = 5. All 10 items are then added, giving a maximum possible score of 50 and a minimum of 10. Up to two “Not Applicable” responses or missing values are allowable and are replaced with the average score for the remaining items. Questionnaires with more than two missing values or “Not Applicable” responses are removed from the analysis. In our study, no questionnaires contained more than two “Not Applicable” answers.

A bivariate analysis will be performed to compare the variables and their values among the two principal groups: control group versus anatomic model group. The choice of variables considered for the multivariate analysis was based on clinical relevance. Categoric variables compared and analyzed using the chi-square and Fisher exact tests. Mann-Whitney *U* tests were used to compare and analyze continuous variables. *P* < 0.05 was considered statistically significant for all tests.

## Results

A total of 304 patients responded to our survey (Table 1). Mean age of the respondents in the control group versus experimental group did not differ significantly (55.0 versus 53.2 years; *P* = 0.321). Civil status of the participating patients was most predominantly married and single in the control group with 38.4% and 33.9% of participants, respectively. In the experimental group, the married and single were also the most common civil status with 32.8% and 47.5%, respectively. No statistical difference between the groups was found with respect to civil status (*P* = 0.121). Medical insurance distribution analysis did not identify any statistical difference (*P* = 0.605). It is worthy to mention that most participants in the study had Medicaid as medical insurance (n = 185). Level of education did not differ among study groups (*P* = 0.401).

**Table 1:**
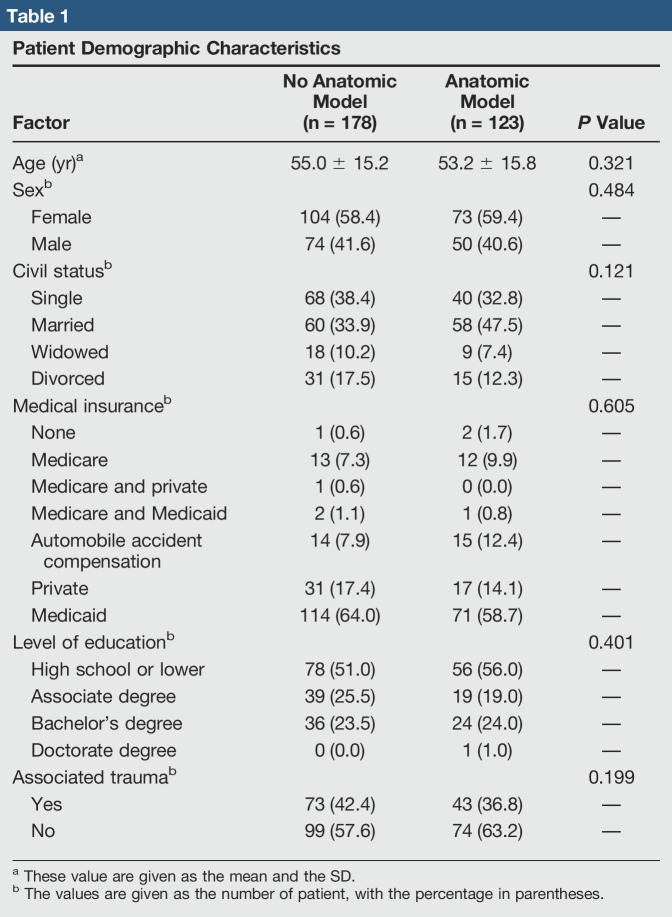
Patient Demographic Characteristics

About half of the participants reports associated trauma for evaluation in the groups (ie, 42.4% control group and 36.8% anatomic group). No statistical difference was found between the control and experimental groups (*P* = 0.199) with regard to the mechanism of injury. In summary, the experimental and control groups are considered similar among the sociodemographic data analyzed. CARE questionnaire median scores (Table 2) for the control group were 44.0 ± 9.0. Scores for the experimental group were 48.0 ± 7.7. Comparison of scores between the two groups did not find any statistical difference (*P* = 0.482).

**Table 2:**
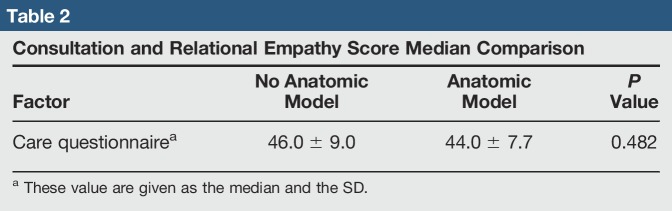
Consultation and Relational Empathy Score Median Comparison

**Figure 3:**
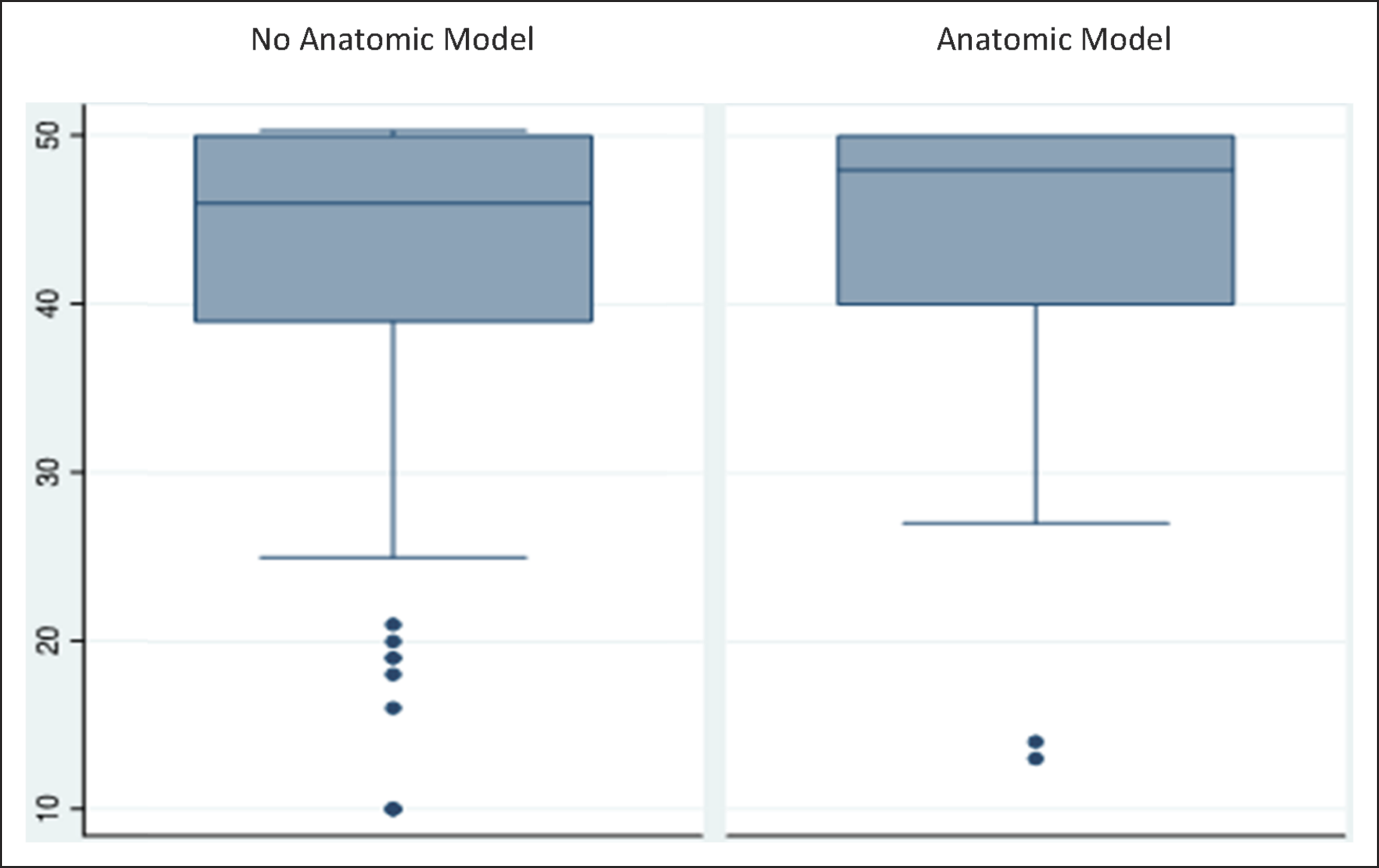
Box plot showing the distribution of Consultation and Relational Empathy scores between the no anatomic and anatomic model groups. No significant differences were found between the groups (*P* = 0.482).

Figure 3 is a box plot showing the distribution of CARE questionnaire scores between the control and anatomic model groups. Lower individual scores were found in the control group, but the overall distribution of scores is similar in both groups. Average score overlapping can be visualized in the box plot.

**Figure 4:**
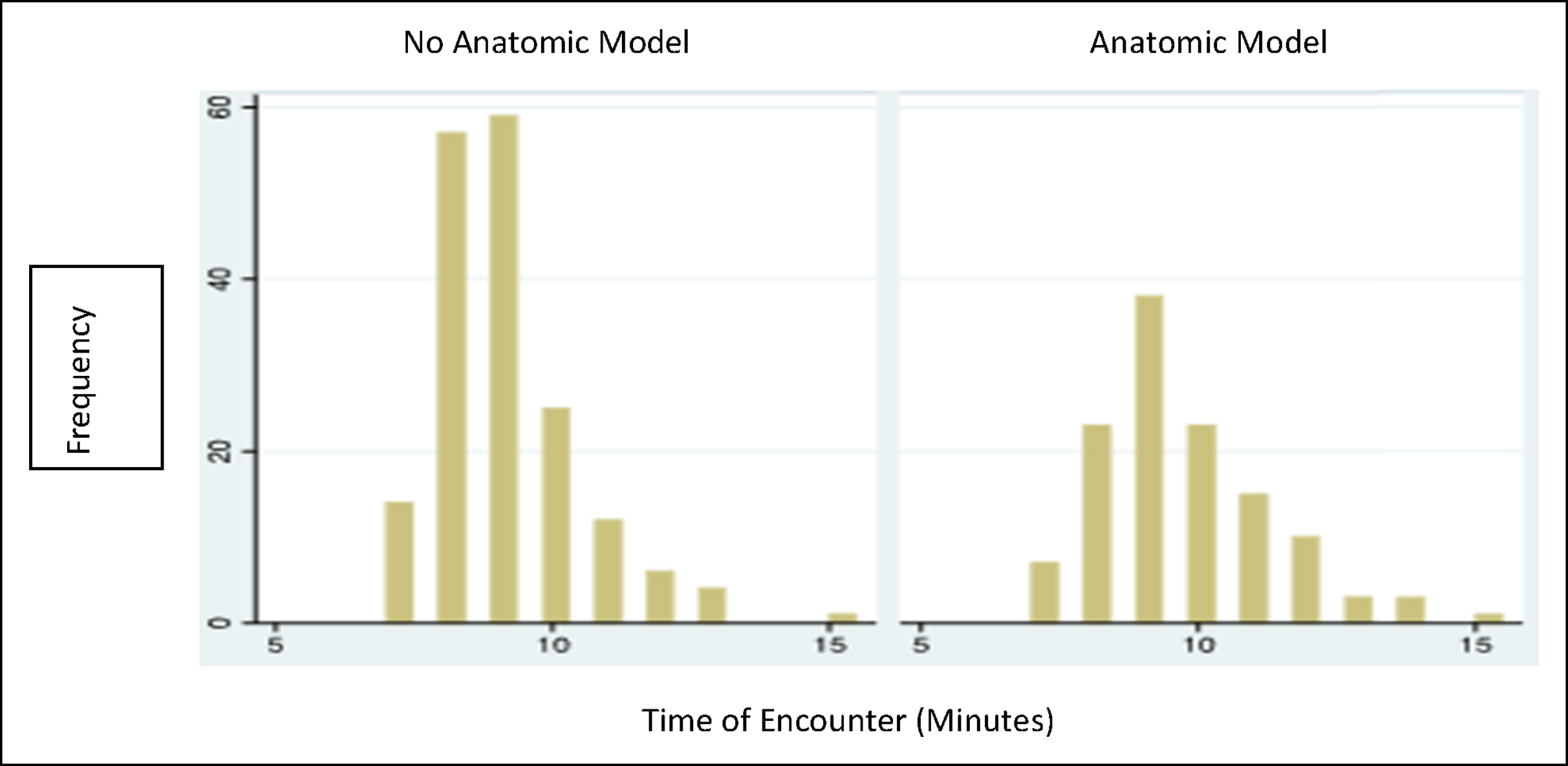
Histogram showing the frequency of the time of encounter between the no anatomic and anatomic model groups. Significant differences were found between the groups (*P* value = 0.0005).

Figure 4 is a histogram showing the difference in distribution of the time of the encounter between the two groups. The anatomic model group encounters on average lasted longer than the control group; no statistical difference was found (*P* < 0.0005).

## Discussion

Analyses of the sociodemographic characteristics did not reveal any significant difference between the control and experimental groups (Table 1). According to the reference CARE Measure normative value (Figure 2), a 50th percentile score correlates with 45.75 median score. The control group's median CARE score is 46.0. CARE Measure scores for the control group followed a similar distribution to the normative values. The experimental group's median CARE score is 48.0. When interpreting this score with the provided normative values, it correlates with a 90 to 95 percentile in satisfaction scores. Statistical analyses between median scores did not find any statistical difference, for which the differences in satisfaction percentile cannot be directly linked to the use of anatomic models.

The encounter time was significantly increased with the use of anatomic models (*P* < 0.005). The study does not analyze the content of the encounter and the difference of distribution of time throughout the encounter. Previous studies have suggested that most time during an encounter with a surgeon was invested in educating and helping patient makes choices about their care.^3^ It would be interesting to measure whether the use of the anatomic model affects the content and more specifically the allotted time in each component of the encounter. Another study reported that primary care physician patients were more satisfied with the longer encounter time but had not found the association with ambulatory orthopaedics.^1^ Our study confirms that the longer encounter time in the orthopaedic appointment does not mean higher empathy perception and satisfaction scores.

In this study, the control and experimental groups did not differ in the level of education between them, but it seems that our participant level of education is skewed to lesser formal education. This phenomenon may limit the generalizability of our study because more formal education may demand different information about their condition. In a recent study, it was suggested that less formal education had lower patient satisfaction scores.^14^ This suggestion opens the possibility of developing a study to compare the use of anatomic models that differ in educational level and how this affects patients' perceived empathy and satisfaction scores with the desired intervention. It is possible that the intervention of the anatomic model proves to be more beneficial to specific populations that share certain characteristics instead of being a measure that may help all patients.

The absence of differences between the groups on our study could be due to various variables. Although patients were not aware of the study during the encounter, participating physicians were. This phenomenon could create an observer bias, which may result in higher scores for the control group because of better performance of the physician during the encounter. Because of our study design, participating investigators had to know about the days the study had to be conducted because they were responsible to make the approach for study participation at the end of the encounter. To eliminate this bias, and possibly find more precise scores, the physician cannot be involved in the approach to participate in the questionnaire.

Validity of the CARE questionnaire could be affected because of our study population. Most of our patients are Spanish speaking. No study has been conducted to validate the translation of the measure in Spanish. Translations of documents do not necessarily translate the spirit of the document in the desired language. Interestingly, our control group scores correlated with the expected normative values of the CARE Measure, but additional studies need to be conducted to validate the questionnaire in Spanish. This phenomenon may limit the power to find any statistical difference with our proposed intervention.

When defining empathy, the opinion of the patient seems to be more important than the physician's perspective of self.^13^ Cultural context has to be an important aspect to improve empathy scores with our patients. In a recent study, the second most common issue for report development was lack of proper communication.^18^ It is therefore important, as suggested by a study, to recognize that patient preferences as per the amount of information desired for their diagnosis may vary.^9^ Every physician should be familiarized with the population they serve and general characteristics that define them. If clinicians do not accurately assess preferences for information, it can result in a conflict between the ideal and actual care received.^10^

Satisfied patients do not necessarily have better outcomes but are more likely to comply with treatments, keep office appointments, and not file complaints or lawsuits.^16^ Pamphlets and informational brochures can supplement, but not replace, effective communication.^4^ Empathy is dependent on a cognitive response, which is the capacity to identify and understand another.^7^ Research suggests that empathy is a skill that can be taught, and surgical curricula in residency should include empathy training.^6^ A provider who is not content with his or her own patient satisfaction scores needs to question himself what measures need to be done to improve.^17^ A previous study specifically demonstrates that surgeons demonstrate weakness in the ability to assess the patient understanding of the situation, discussing risks and uncertainties associated with their care.^3^ Given the multiple variable that seem to affect patient satisfaction scoring, it seems that physicians will need to be actively studying the characteristics of their population to identify strategies to improve satisfaction scores.^17^

To our knowledge, this is the first study that directly addresses the use of anatomic models during the medical encounter and how these affect the empathy perception and patient satisfaction scores in the orthopaedic population. Anatomic models are just one of the many tools that may improve communication, empathy, and patient satisfaction. Although there may be various nonmodifiable patient factors, such as age and geographic location,^11^ there are also modifiable variables that can be addressed to improve patient satisfaction. Previous research has reported that only 53% of the patients treated by orthopaedic surgeons disclosed real concerns about surgery.^8^ Consciousness about the topic may help the orthopaedic surgeon to improve his interaction with patients. Practical solutions such as the use of question list made pre-encounter to remind patients to ask important questions may make significant differences in the communication.^12^ All aspects of the encounter need to be addressed to optimize patient satisfaction.

## Conclusion

The use of anatomic models did not confer a statistical advantage to improve the perceived empathy and patient satisfaction during an initial appointment. The encounter time was increased with the use of anatomic models. As orthopaedic surgeons, we are responsible for finding strategies to improve the communication skills with our patients. Dedicating time to understand the variables that affect patient satisfaction may help improve patient-physician interaction. Further investigation should be considered to use other strategies to provide better care for our patients.
